# Grape (*Vitis vinifera* L.) Seed Oil: A Functional Food from the Winemaking Industry

**DOI:** 10.3390/foods9101360

**Published:** 2020-09-25

**Authors:** Maria E Martin, Elena Grao-Cruces, Maria C Millan-Linares, Sergio Montserrat-de la Paz

**Affiliations:** 1 Department of Cell Biology, Faculty of Biology, Universidad de Sevilla, C/ Profesor Garcia Gonzalez, s/n, 41012 Seville, Spain; mariamartin@us.es; 2 Department of Medical Biochemistry, Molecular Biology, and Immunology, School of Medicine, Universidad de Sevilla, Av. Dr Fedriani, 3, 41071 Seville, Spain; egrao@us.es; 3 Department of Food & Health, Instituto de la Grasa, CSIC. Ctra. de Utrera Km. 1, 41013 Seville, Spain; mcmillan@ig.csic.es

**Keywords:** wine, grape seed oil, functional food, fatty acid, nutraceutical

## Abstract

Wine production is an ancient human activity that generates several by-products, which include some constituents known for their potential in health care and for their role in the food or cosmetic industries. Any variety of grape (*Vitis vinifera* L.) contains nutrients and bioactive compounds available from their juice or solid parts. Grape seed extract has demonstrated many activities in disease prevention, such as antioxidant effects, which make it a potential source of nutraceuticals. Grape seed is a remarkable winery industry by-product due to the bioactivity of its constituents. Methods for recovery of oil from grape seeds have evolved to improve both the quantity and quality of the yield. Both the lipophilic and hydrophilic chemicals present in the oil of *V. vinifera* L. make this wine by-product a source of natural nutraceuticals. Food and non-food industries are becoming novel targets of oil obtained from grape seeds given its various properties. This review focuses on the advantages of grape seed oil intake in our diet regarding its chemical composition in industries not related to wine production and the economic and environmental impact of oil production.

## 1. Introduction: An Overview of Wine to the Past from the Present

Wine is an alcoholic beverage that has been consumed for many years. The beginning of winemaking has been dated to 5400–5000 BC in the southern Caucasus region. From here, wine grape (*Vitis vinifera* L.) growing spread to Mediterranean countries, where Greek philosophers and European healers noted the healthy benefits of wine products. Consumption of wine became regular amongst Romans, whose Empire helped to broaden winemaking in Europe. Some data also place grape use in Egypt back 6000 years. Socioreligious implications may explain the diverse implementation of wine intake and production in Christian or Islamic regions, as well as its expansion to the Americas [1,2].

Nowadays, the winemaking industry and global consumption of wine are huge socio-economical markets [3,4]. A production of 250–300 million hectoL/year during the last two decades (Table 1) correlates with the environmental impact of this activity and the promising use of wine-derived products in other industries and medicine [5].

Wine is the result of the chemical fermentation of must or grape (*Vitis vinifera* L.) juice, carried out by yeasts that consume grape sugars and produce ethanol and CO_2_. Up to 500 substances are produced though the processes of winemaking, storage, and maturation of the beverage. They are responsible for some of the characteristics and sensory features of wine. Regarding phenolic constituents, or polyphenols, also present in fruit, grains, and vegetables, data from several studies suggest that they have healthy properties [1,6,7,8]. Resveratrol, a non-flavonoid present in trace quantities, is a natural antioxidant and anti-inflammatory compound, known to play a key role in immunoregulation, metabolic syndrome, cardiovascular, neuroprotection, and cancer treatment [9,10,11,12,13,14,15,16,17,18,19,20]. It has also received attention due to its antimicrobial activity against bacterial, fungal, and viral species in vitro [21,22,23,24].

In the winemaking industry, grapes as well as grape residues (pomace or marc), including seeds and lees, are interesting products considering the health benefits of their components. By-products such as oil seeds or pomace yeasts have been reported to be natural sources of bioactive [25,26,27,28,29]. Therefore, wineries have broadened their economic and environmental impact through the use of natural bio-products.

## 2. Grapes, Seeds, and Seed Extracts: Natural Sources of Nutrients and Bioactive Compounds from Winemaking Industry

**Figure 1:**
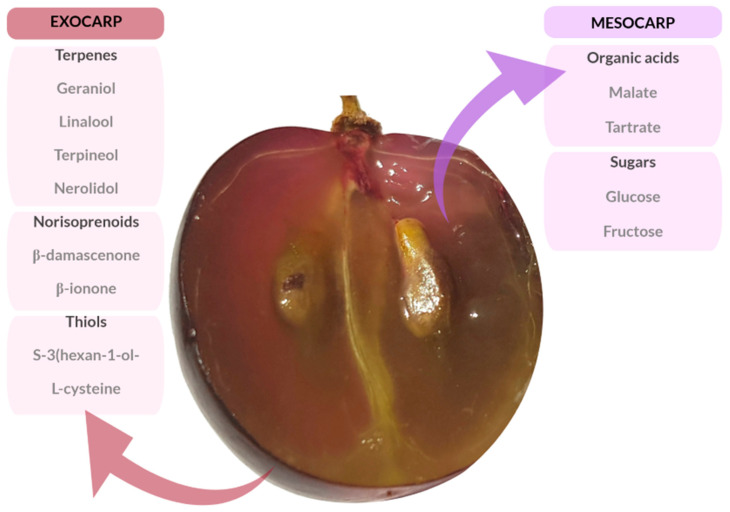
Grape layers: exocarp and mesocarp. The exocarp is the outer layer of the fruit with a pulp rich in terpenes, norisoprenoids, and thiols. The mesocarp is a tissue rich in organic acids and sugars.

Grapes are one of the most consumed fruits in the world, mostly in the form of juice and wine. Over 1000 species of red and white grapes are extensively cultivated around the world, and their composition influences the wine chemistry [1]. Generally, grapes have juice, pulp, skin, seeds, and stem. Regarding chemical components, the two important layers are (Figure 1): exocarp, which includes terpenes, norisoprenoids, and thiols; and mesocarp, with organic acids and sugars.

Berries are plentiful in sugar, whereas phenolic compounds are inexpensive sources of natural antioxidants and antimicrobial agents that are obtained from grape wastes during winemaking production [30,31,32]. Grape berry phenolics contribute to organoleptic properties of wine, though their composition depends on grape variety and harvest conditions, and even on grape maturity degree. Skin is enriched in tannins and non-flavonoid stilbenes (resveratrol), while non-flavonoid hydroxycinnamic acids are the most abundant in flesh. By contrast, seeds contain mainly flavan-3-ols and many non-flavonoids, including those mentioned in skin and flesh, with a total phenol content in seed 10 times greater than in the peel [33,34]. As a matter of fact, grape seeds and the bioactive oil from them are the main topic of this review.

Grape seeds are a relevant part (10–12%) of the solid residues created from the winemaking process [35]. However, vinification protocols may affect the type of seed obtained in this marc or pomace. Thus, white winemaking generates pomace directly, whereas red wine production includes a maceration period in alcohol prior to obtaining marc; distillation of pomace may lead to grape seed production [36,37]. As already mentioned for berries, phenols and other components of grape seeds may be of interest from a nutraceutical or health-prevention perspective; pomace extraction has been studied to improve phenols yield, with several maceration protocols being developed [38,39]. In line with this, Guaita et al. analyzed the phenolic composition of fermented and unfermented pomaces, reporting no considerable differences after processing. Bordiga et al. explored the oligosaccharides from grape marc distillation as probiotic products [40,41].

The phenols in *Vitis vinifera* L. seeds provide health benefits and proanthocyanidins, also known as condensed tannins, play a potential nutraceutical role. The antioxidant and free radical scavenging abilities of phenols have been demonstrated both in vivo and in vitro, protecting more than vitamins E or C and β-carotene [42,43,44]. Other activities—such as anti-inflammatory [45], antimicrobial [46], antiulcer [47], and anticancer—have also been reported [48,49,50]. However, the polymerization of the molecules is known to affect their bioavailability, so further research is needed to clarify the potential of tannins as a dietary supplement for humans. [51]. Tannins are rarely present in the oil obtained from *Vitis* sp. seeds, but are plentiful in polyunsaturated fatty acids, as we discuss in depth in this review. Studies regarding whether the oil extraction method may affect the antioxidant components of grape seed oil should be undertaken [35,52].

Of grape seed extracts, antioxidant and antimicrobial activities can be noted due to their phenolic components [53,54,55]. Additionally, some evidence shows that grape seed extract may provide anti-cancer chemoprevention [56,57,58,59,60,61] and cardio-protection [62]. Hao et al., studied the possible effect regarding hypertension and vascular remodeling to prevent stroke in rats [63]. Vinson et al. tested extract components in a hamster atherosclerosis model [64]. Bijak et al., working with in vitro models, first suggested that extracts could act as a nutraceutical in the prevention of thrombosis [65]. Under ischemic conditions, neuroprotective effects, reducing brain damage, coupled with an anti-apoptotic activity and proteome preservation have been reported in animals pre-treated with grape seed extracts [66,67]. The role of grape seed extracts in attenuating inflammation and delaying the development of Alzheimer’s or acting as a neuroprotection agent in Parkinson’s disease have been reported [68,69]. In rats and mice, grape seed extract was suggested to improve conditions associated with metabolic syndrome [70,71,72]. Grape seed extracts are also beneficial in bone healing [73], skin disorders [74,75], and show a photoprotector activity [76]. Despite these promising results, human consumption of grape seed extract is a controversial topic and not many studies have reported clear conclusions. Only a few trials in animals or humans proved that a grape seed extract may be an efficient source of antioxidants [77,78,79].

Considering their antioxidant and antimicrobial extracts activities, phenolic-rich grape seed extracts may also be used in the food industry as natural additives to prevent microbial growth and lipid oxidation, thereby increasing product quality, safety, and shelf life [80,81].

## 3. Grape Seed Oil: A Novel Functional Food

The curative effects of grape seed oil have been presented in the literature since the 14th century in Spain, when an Arab doctor suggested that Ferdinand IV, King of Castile and Leon in the Iberian Peninsula, use it for the treatment of skin problems. The king decided to protect its composition and named it “royal oil” or “oil of the throne” [2]. Nowadays, recovery of oil from grape (*V. vinifera* L.) seeds is probably the main application due to the huge amount of seeds produced worldwide and because of the health benefits of grape seed oil intake in our diet, as well as its potential use in other non-food industries.

**Figure 2:**
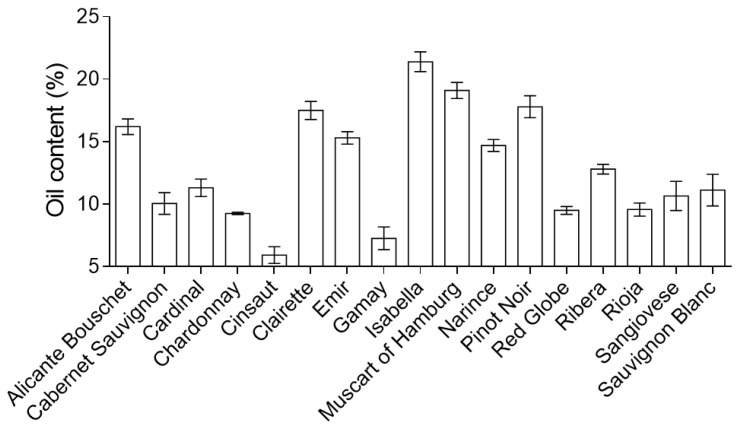
Oil content in grape seeds of wine varieties. Content of oil is given in grams per 100 g of dry weight of the seeds [82,83,84,85,86,87,88,89,90,91,92].

As there is more than one type of grape, the proportion of oil obtained varies with the type of grape. The proportion of oil varies between 20% obtained from sweet white grape seeds and 6% obtained from some black varieties (Figure 2) [82,83,84,85,86,87,88,89,90,91,92].

### 3.1. Grape Seed Oil: Extraction Processes

Grape seed oil has been extracted for many years using organic solvents, usually hexane (Soxhlet method), or pressing mechanical techniques. Solvent extraction is the more expensive method as it requires a purification final step due to the toxicity of the hexane and because it removes pigments and waxes, generating a dark and viscous product. Both hexane and pressing protocols have a high oil yield but the high working temperature is a limitation to preserving the quantity and quality of the bio-compounds obtained [2,35,93].

Cold pressing extraction became an alternative method to obtaining quality oil, solving the temperature problem, although the yield is lower, unless the protocol includes an enzymatic pre-treatment, which alters the cell walls and improves oil extraction. Nevertheless, considering the lack of solvents, the oil obtained by pressing is a much safer source of health-beneficial phytochemicals and may even result in higher fatty acids and tocopherol contents in the final oil composition [94,95]. Mechanical expeller pressing techniques have also been tested to obtain healthy grape and other oils. The operating conditions of the expeller, such as temperature and speed, may improve time and oil yield, respectively, whereas sample moisture seems to reduce production [96]. When compared to animal fats as a substitute for dietary fats, fruit seed oils extracted with solvents or through expeller pressing show cardioprotective activity, lowering cholesterol risk ratios, regardless of the extraction method [97].

Eco-friendly edible oil extraction strategies attempt to overcome the remaining limitations regarding the use of chemicals and the temperature of the extraction process. In this sense, hot water extraction and supercritical fluid extraction (SFE) use fluids and CO_2_, using seeds or other parts of the plants. Some data demonstrate that for *Cinnamomum*, superheated water extraction yields high quality oil, reducing production time and costs [98], being also useful for extracting phenols and oil from peppermint simultaneously [99]. SFE with CO_2_ is a green and low-cost alternative that produces a high-quality product compared to mechanical pressing, and though yield is lower than that of hexane-extraction, as with cold-pressing, it may improve using an enzymatically pre-treated seed [100,101]. SFE has been postulated to be a suitable protocol for recovering lipophilic antioxidants from wine wastes [93].

Other oil extraction methods include pressurized liquid extraction (PLE) [102,103], microwave-assisted extraction (MAE), and ultrasound-assisted extraction (UAE) [104,105,106]. MAE uses nonionizing electromagnetic waves that are transformed to thermal energy, whereas UAE takes advantage of negative pressure after ultrasound treatment. Both techniques look for damage in cell walls to facilitate the extraction [93]. Using PLE, MAE, and UAE, lipid yields from *Echium* seeds were similar to those extracted with hexane [107]. Some findings demonstrated that the MAE protocol is suitable for the extraction of phenolic antioxidants from grape seeds [108].

In this review, we focus on *Vitis vinifera* grape seeds that show the best extraction oil yield using SFE compared to Soxhlet or MAE and UAE. The fatty acid composition is not remarkably affected by the extraction protocol, though antioxidant activity appears to be benefited by ultrasound technique [93]. Little was found in the literature concerning differences in grape seed oil compositions depending on the seed extract method, so further research is needed to optimize the bioactive compound production using environmentally friendly protocols [35].

### 3.2. Grape Seed Oil: Chemical Composition

**Figure 3:**
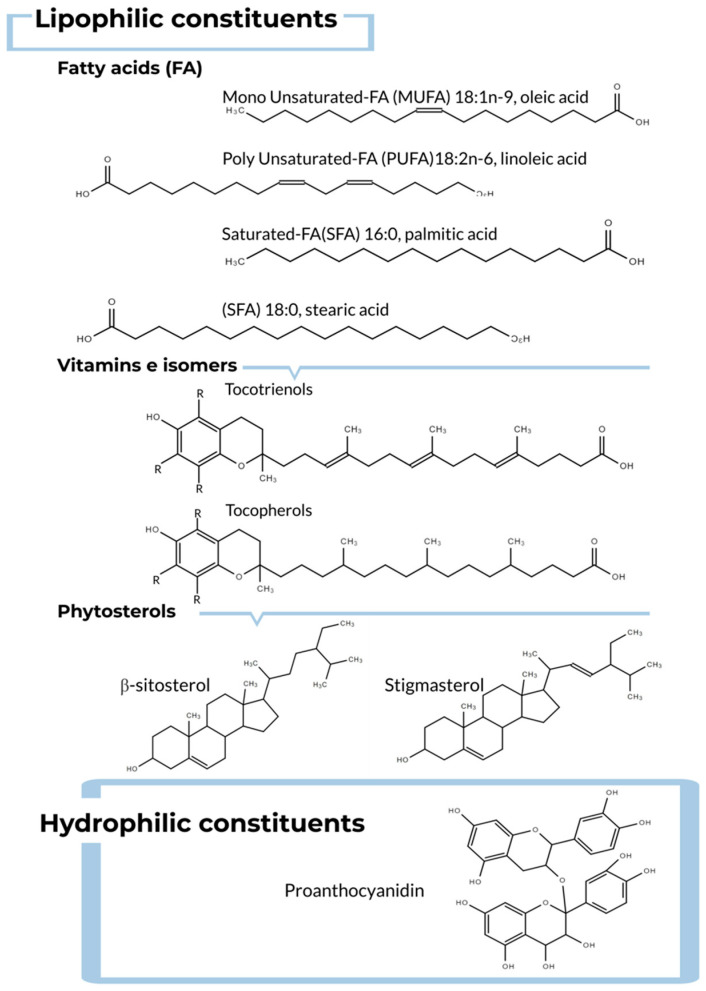
Chemical structures of main compounds in grape seed oil.

Besides grapes, seeds, and extracts, grape seed oil is a winemaking by-product of interest regarding its bioactivity in terms of disease prevention or treatment. In grape seed composition, around 6–20% is oil [82,83,84,85,86,87,88,89,90,91,92] and its chemical composition depends mainly on the degree of maturation of the seeds employed, the grape variety and many environmental cultivation conditions and to a lesser degree on the seed extraction protocol, as already mentioned [35,109]. Nevertheless, in contrast to berries, seeds, or extracts, most of the grape seed oil’s constituents are lipophilic molecules, which also include several important biocompounds (Figure 3).

We considered three groups of molecules included in the lipophilic total constituents of grape seed oil. Fatty acids (FAs) are the most abundant, vitamin E isomers are also present, and the phytosterol content is the lowest in this group.

Firstly, unsaturated fatty acids (UFAs) comprise almost 90% of the total fatty acid composition in grape seed oil. Among them, cold-pressing techniques yield around 65–75% of the polyunsaturated FA (PUFA) linoleic acid (LIA) (C18:2n-6) and 20–40% of oleic acid (C18:1n-9), a monounsaturated FA (MUFA), depending on the variety of seed analyzed. Saturated fatty acids (SFAs) are present in low quantities, around 10% [82,83,109,110,111]. The fatty acids of edible oils are prone to oxidation; thus, the lipid composition of grape seed oil affects its shelf-life. As we discuss in this review, this is important when considering vegetable oils for human intake regarding the bioactivity of their components [112]. Table 2 shows the FA composition of various oils, including grape seed oil (*V. vinifera* L.). The data Table 2 show that grape seed oil is plentiful in LIA, with the highest concentration of this PUFA, second only to safflower oil. This is important as LIA promotes cardiovascular health in animal models [113]. In addition, this PUFA may modulate odor, taste, and shelf-life of the grape seed oil depending on the concentration and the oxidative stability of their molecules. Together with the previous data regarding the abundance of LIA in grape seed oil, this oil stands out not only as a potential nutraceutical but also as a modulating chemical in the oil properties. Oleic acid is the second most abundant FA in grape seed oil. However, as shown in Table 2, this MUFA is not plentiful in comparison to other edible vegetable oils, with less than half the concentration of peanut, almond, and rapeseed oils. Nevertheless, oleic acid consumption is high in the Mediterranean diet, whereas oils enriched in oleic are rarely present in Mediterranean intake. Additionally, oleic acid acts as an oxidative regulator of grape seed oil, as reported for LIA. Thus, oil from grape seeds, among plant oils, appears to be relevant for human intake and oil preservation. Regarding SFAs present in grape seed oil, the quantification in Table 2 shows expected values around 10%. Edible vegetable oils, in comparison, have similar or at most double concentrations of these lipids, except for a percentage close to 90% of saturated fatty acids in coconut oil; this oil is often used in cooking. Palmitic acid is present in higher concentrations than stearic acid in all the oils tested except for coconut oil [2,35,109,114,115,116,117,118].

In the second group of lipophilic molecules, up to 50 mg of vitamin E is present in every 100 g of grape seed oil depending on the growing circumstances and the kind of grape analyzed. Vitamin E is known to have remarkable antioxidant activity, which makes it beneficial for human health [35,82,109]. Many seed oils contain vitamin E isomers, tocopherols, and tocotrienols in different concentrations, as shown in Table 3, where, in general, the tocotrienols content is higher compared with tocopherols. It was reported that the presence of both isomers is also affected by the degree of maturation of the grape seeds and berries [119]. Data showed that tocopherol levels decrease during seed maturation, whereas tocotrienols increase in content with seed growth, even exceeding tocopherols content. This may explain the wide range of the data in Table 3. As indicated, vitamin E concentrations are influenced by harvest conditions because they influence vegetation periods and then the degree of maturation of the seeds used to extract oil [119]. Closer inspection of Table 3 indicates that the α and β homologues are more present than γ-tocopherols. Regarding tocotrienols, γ-isomers are more abundant. γ-tocopherol is known to be an important antioxidant constituent, not only because it is rarely found in other oils, but also because it should be consumed with the diet since it cannot be produced by mammals [2,82,92,120,121,122]. Finally, all the tocopherol forms must be protected from light and air, which is challenging for both extraction and analyses processes. Therefore, to record tocopherols measurements, sample preparation is critical to avoid degradation; direct examination can be performed after diluting the oil in an organic solvent before quantification, for example using HPLC [121].

Finally, phytosterols are also lipophilic molecules present in grape seed oil constituents, at around 2–11 mg/g oil. As already mentioned for other constituents, the sterols concentration in seed oil is affected by harvest conditions and the oil extraction method. Table 4 shows the contents of the main phytosterols in grape seed oil. The highest concentration found was β-sitosterol, up to 65%, far from the next, stigmasterol, at around 10%. In line with this data, in plants, β-sitosterol, campesterol, and Δ5-stigmasterol are generally the most abundant sterols. The biological importance of phytosterol is due to its antioxidant activity as well as its role in cholesterol metabolism. In particular, β-sitosterol together with polyphenols from the winery industry have shown this cardioprotective activity in vitro, preventing the release of pro-inflammatory and pro-atherogenic molecules [35,109,119].

In the oil total composition, grape seed oil contains a large number of hydrophilic constituents as well. Many of them are phenolic compounds (60–70%) that—as already explained for berries, seeds, and extracts—are known as bioactive chemicals regarding their antioxidant activity. Although the total phenolic constituents in grape seed oil are scarce, as previously explained for seeds, extraction protocols can be optimized to increase antioxidant compounds yields. Data obtained so far suggest that pressing mechanical extraction is suitable for recovering polyphenols from residues during oil production [123,124].

### 3.3. Grape Seed Oil: Nutraceutical Activities

This review focused on oils extracted from grape seeds, which are a component of winery industry by-products together with berries, seeds, and extracts, which have become of interest regarding the bioactivity of some of their components. Therefore, grape seed oil has been reported as a suitable dietary supplement that may prevent or improve physiological disorders related to chronic diseases [35,109]. Notably, many questions related to bioavailability, dose response, and side effects in humans are still to be answered [125,126]. Nevertheless, we will address the main nutraceutical activities with the responsible oil components.

#### 3.3.1. Antioxidant Activity

Phenolics have been highlighted as natural antioxidant molecules present in grapes, with higher contents in grape seeds and extracts. In contrast, hydrophilic phenols are minor compounds of grape seed oil [82,127] but, as previously mentioned, oil extraction protocols can improve conditions to increase phenol yields in the short term [123,124], highlighting the importance of producing new natural chemicals able to alleviate oxidative stress conditions, scavenge free radicals, inhibit lipid oxidation, or reduce hydroperoxide formation in contemporary diseases [35]. Among phenolic compounds in grape seed oil, gallic acid, cathecin, epicathecin, procyanidins, and proanthocyanidins or condensed tannins are known for their antioxidant bioactivity [109]. Vitamin E and phytosterols—the second- and third-most abundant lipophilic compounds in grape seed oil composition after fatty acids, respectively—are also known to have antioxidant activity [128]. Some data also showed that vitamin E can delay aging and the outcome of chronical diseases [82].

#### 3.3.2. Anti-Inflammatory Activity

Both hydrophilic and lipophilic components of grape seed oil contribute to recovery from the inflammation processes that occur during many chronical diseases. In animals, inflammatory parameters, such as cytokines, can be quantified to test the pathological conditions before and after grape seed oil intake [35]. Among other mechanisms, phenolic compounds are known to modulate anti-inflammatory gene expression, influencing several cellular pathways including arachidonic acid release, cytokines production, or NO (nitric oxide) synthase activity [129]. Tocotrienols, isomers of vitamin E, may influence adipose inflammation related to obesity per evidence in cell lines [130]. Inflammation conditions also affect insulin resistance and some studies demonstrated the beneficial influence of grape seed oil in affected humans according to the presence of phenolics and tocotrienols [35,131,132]. Included in phytosterols, β-sitosterol also seems to be a prevention factor for the liberation of modulators of pro-inflammation conditions by oxidized low-density lipoprotein (LDL)-stimulated macrophage cells on oxidative stress and eicosanoid synthesis [35,133,134].

Recent data point to linoleic acid as an anti-inflammatory natural agent for mammalian cells [135]. LIA is the most abundant fatty acid in the grape seed oil composition, which also contains many lipophilic chemicals. Thus, it is necessary to conduct more studies to deeply analyze the anti-inflammatory activity of LIA from grape seed oil as a potential nutraceutical suitable for human consumption.

Both antioxidant and anti-inflammatory activities of grape seed oil have been reported as the basis of another neuroprotective and hepatoprotective behavior of grape seed oil tested in animals [136,137]. In human cells, grape seed oil hydrophilic components have already been shown to attenuate oxidative and inflammation conditions [138].

#### 3.3.3. Antimicrobial Activity

The antimicrobial activity of grape seed oil has been reported against certain pathogens, such as *Staphylococcus aureus* and *Escherichia coli* [109,139]. In detail, phenolic compounds, mainly resveratrol, plays a key role in causing oxidative damage to the plasma membranes of the bacteria. Growing interest in this topic points to grape seed oil or its components as coadjutants in antimicrobial therapies.

#### 3.3.4. Antitumoral Activities

Previous studies findings related natural polyphenols from seeds to a promising anticancer activity. From the experiments conducted to date, both with cells and in animal models, these compounds may either impair gene expression or signaling pathways, affecting intracellular events that occur in affected or healthy cells [140,141,142]. Some data also indicate antitumor effects of tocotrienols from grape seeds [128]. More recently, dietary plant phytosterol supplementation has been considered in anticancer experiments [143,144,145].

Regarding clinical applications, grape seed oil has been used as the basis of lipid nanocarriers to optimize the therapeutic efficiency of antitumor drugs and therefore its toxicity [146].

#### 3.3.5. Protective Activities

Many studies have focused on the cardioprotective potential of grape seed oil. In vitro lowering of platelet adhesion has been reported [109] and intake in animals was already tested for its effects in lipid profiles [35]. In rats, it has been postulated that this grape seed oil lowers cholesterol levels [147]. Some studies pointed to a role of polyphenols and phytosterols as the natural nutraceuticals support therapies in cardio pathologies [148]. Nevertheless, the effect of grape seed oil in lipid human profile requires further research [109]. In addition, it has been highlighted that linoleic acid, plentiful in grape seed oil, may promote cardiovascular health in animal experiments, showing potential as a food supplement [135].

Promising results have also been published regarding the potential neuroprotective activity of grape seed oil as a diet supplement in animals with Alzheimer’s disease [149].

#### 3.3.6. Dietary Activities

Diets for chickens supplemented with grape seed and oil have been tested, affecting cholesterol and lipid content [150]; promising results for cholesterol transport in rats have been reported [151]. Grape seed oil supplementation in diets of animals for human consumption has been analyzed in various studies. Concerning the improvement of pork meat, reduction of fat levels was reported [35]. In ruminant animals, scarcely affecting animal digestibility, antioxidant status and lipid profile were tested as parameters to consider for further human ingestion [152].

Concerning human consumption, vegetable oils are an alternative to animal fats in terms of health maintenance and chronic diseases prevention as a cardioprotective agent. Grape seed extracts have already been used in combination with oil supplements to alleviate the hyperlipidemic postprandial conditions [153]. Grape seed oil LIA is essential in the human diet, as we are not able to synthesize it, and it is a precursor of other fatty acids, then we need a supplement to this PUFA. The dietary intake of linoleic acid (n-6) must consider the ratio of fatty acids n-6/n-3 (4–5/1 recommended) to maintain the balance of fatty acids and avoid inflammation processes [154,155]. Diets supplemented with excessive n-6 fatty acids have been related to chronical diseases via oxidative stress as a consequence of intracellular damage caused by the oxidation of the lipid chains [35,156]. There is evidence that the fatty acids contained in grape seed oil may be pro-oxidative compounds [157,158]. Experiments with animals comparing different types of oils have not provided definitive conclusions. In summary, further research is required to determine the detailed bioactivity and the recommended doses for human consumption of LIA [114].

### 3.4. Grape Seed Oil: Other Applications of Interest

The interest of the food industry in vegetable oils is also increasing. Meat products were improved in terms of quality when adding grape seed oil instead of animal fats [159]; the effect was significant when the oil was previously emulsified with rice bran fiber [160]. Popular products such as sausages were found to be healthier when grape seed oil was added [161]. Grape seed oil is also becoming popular as a culinary oil due to its organoleptic properties. Considering its high smoking point, it can be used to fry food. As it emulsifies well, this oil can be included in dressings and sauces, such as mayonnaise. The flavor of the food is not affected because grape seed oil’s flavor is unobtrusive.

Regarding the use of grape seed by-products in non-food industries, pharmaceutical and cosmetics manufacturers also take advantage of the presence of bioactive compounds in their composition [162]. Grape seed proanthocyanidins have shown a preventive photo-carcinogenesis effect [163]. The antioxidant activity of many constituents of grape seed extract show skin protective activity [164]. Both in animals and humans, grape seed oil shows wound-healing activity and diminishes scars [2,165]. In the cosmetics industry, grape seed oil might be used as an ingredient in skin moisturizer products, with a soft texture, leaving no residues when applied, and lacking allergic reactions. Loss of linoleic acid seems to be one of the reasons for water loss in the skin; thus, grape seed oil reverts the skin to standard conditions. Skin tightening, mostly to reduce swelling, is another effect of its astringent property that makes grape seed oil a common ingredient in cosmetic products. Not only does linoleic acid improve skin health in general, it also strengthens cell membranes and shows antioxidant and anti-inflammatory activities. This is related to the suitability of grape seed oil use for acne problems. Prior studies have proved that regular application of cosmetic products containing grape seed oil, alone or mixed with other vegetable oils, are recommended for dark circles under the eyes or androgenetic alopecia [2,166].

Finally, grape seed oil appears to be a promising competitor for fossil sources of fuel due to the presence of unsaturated fatty acids in its constituents. Biodiesel production, including refining after extraction, is an economic and environmentally friendly alternative in regions with high volumes of wine production [162,167,168].

## 4. Conclusions

As a conclusion, the winery industry creates a large amount of waste, like seeds from grapes. Many of those by-products can impact both the prevention and onset of diseases. Regarding grape seed oil, some constituents show remarkable antioxidant and anti-inflammatory activities. Essential fatty acids—like linoleic acid, vitamin E, and phytosterols, as well as hydrophilic phenols—appear to be promising not only as nutritional but also as therapeutic compounds. Many of them are also under experimental trials to explore their anticancer properties. Other non-food industries may also benefit from grape seed oil, including pharmaceuticals and cosmetics. Considering the vast extent of grape crops, the use of winemaking-related compounds has also become an environmental impact topic, added to the economic worldwide market.

## Figures and Tables

**Table 1:** Wine production by top 15 wine-producing countries [5]. Data are expressed in volume (thousands of hectoliters).

Country	2014	2015	2016	2017	2018
Italy	44,200	50,000	50,900	42,500	54,800
France	46,500	47,000	45,200	36,400	49,100
Spain	39,500	37,700	39,700	32,500	44,400
United States	23,100	21,700	23,700	23,300	23,900
Argentina	15,200	13,400	9400	11,800	14,500
Chile	9900	12,900	10,100	9500	12,900
Australia	11,900	11,900	13,100	13,700	12,900
Germany	9200	8800	9000	7500	9800
South Africa	11,500	11,200	10,500	10,800	9500
China	11,600	11,500	11,400	11,600	9300
Russia	4800	5600	5200	6300	6500
Portugal	6200	7000	6000	6700	6100
Romania	3700	3600	3300	4300	5100
Hungary	2400	2600	2500	3200	3600
Brazil	2600	2700	1300	3600	3100
Rest of the world	27,100	29,800	29,900	26,100	26,800
World	270,000	277,000	273,000	249,800	292,300

**Table 2:** Fatty acids (FAs) composition (%) of vegetable oils [114,115,116,117,118].

FAs	SAF	GRP	SIL	HMP	SFL	WHG	PMS	SES	RB	ALM	RPS	PNT	OL	COC	EPO
C6:0	nd	nd	nd	nd	nd	nd	nd	nd	nd	nd	nd	nd	nd	0.52	nd
C8:0	nd	0.01	nd	nd	nd	nd	nd	nd	nd	nd	nd	nd	nd	7.6	nd
C10:0	nd	nd	nd	nd	nd	nd	nd	nd	nd	nd	0.01	nd	nd	5.5	nd
C12:0	nd	0.01	0.01	nd	0.02	0.07	nd	nd	nd	0.09	nd	nd	nd	47.7	nd
C14:0	0.10	0.05	0.09	0.07	0.09	nd	0.17	nd	0.39	0.07	nd	0.04	nd	19.9	nd
C15:0	nd	0.01	0.02	nd	nd	0.04	nd	nd	nd	nd	0.02	nd	nd	nd	nd
C16:0	6.7	6.7	7.9	5.6	6.2	17.4	13.1	9.7	20.0	6.8	4.6	7.5	7.5–20	nd	6.3
C17:0	0.04	0.06	0.06	0.05	0.02	0.03	0.13	nd	nd	0.05	0.04	0.07	nd	nd	nd
C18:0	2.4	3.8	4.5	2.68	2.8	0.7	5.7	6.5	2.1	2.3	1.7	2.1	0.5–5	2.7	1.9
C20:0	nd	0.16	2.6	2.5	0.21	nd	0.47	0.63	nd	0.09	nd	1.01	0.43	nd	0.3
C22:0	nd	nd	nd	0.4	nd	nd	nd	0.14	nd	nd	nd	nd	0.15	nd	0.1
C16:1 (n-7)	0.08	0.2	0.05	0.31	0.12	0.21	0.12	0.11	0.19	0.53	0.21	0.07	0.3–3.5	nd	nd
C17:1 (n-7)	nd	nd	0.03	nd	nd	nd	nd	nd	nd	nd	nd	nd	nd	nd	nd
C18:1 *cis* (n-9)	11.5	14.8	20.4	11.9	28.0	12.7	24.9	41.5	42.7	67.2	63.3	71.1	55–83	6.2	6.9
C18:1 *trans* (n-9)	nd	nd	nd	nd	nd	nd	nd	nd	nd	nd	0.14	nd	nd	nd	nd
C20:1(n-9)	nd	0.40	0.15	1.44	0.18	7.91	1.08	0.32	1.11	0.16	9.1	nd	0.30	nd	0.6
C18:2 *cis* (n-6)	79.0	74.2	63.3	55.1	62.2	59.7	54.2	40.9	33.1	22.8	19.6	18.2	3.5–21	1.6	73.9
C18:3 (n-3)	0.15	0.11	0.88	16.7	0.16	1.2	0.12	0.21	0.45	nd	1.2	nd	<1	nd	nd
C18:3 (n-6)	nd	nd	nd	3.4	nd	nd	nd	nd	nd	nd	nd	nd	nd	nd	9.2
SFAs	9.3	10.6	15.1	11.2	9.4	18.2	19.6	16.9	22.5	9.3	6.3	10.7	8–26	92.1	nd
MUFAs	11.6	14.9	20.7	13.3	28.3	20.9	26.1	42.0	44.0	67.9	72.8	71.1	53–87	6.2	nd
PUFAs	79.1	74.3	64.2	75.5	62.4	61.0	54.3	41.2	33.6	22.8	20.9	18.2	3–22	1.6	nd
n-3 PUFAs	0.2	0.2	0.9	0.4	0.2	1.2	0.1	0.2	0.5	0.0	1.2	0.0	1.6	0.0	nd
n-6 PUFAs	79.0	74.7	63.3	62.4	62.2	59.7	54.2	40.9	33.1	22.8	19.6	18.2	16.4	1.6	nd

Note: nd, FAs were not detected; SAF—safflower; GRP—grape; SIL—*Silybum marianum*, HMP—hemp; SFL—sunflower; WHG—wheat germ; PMS—pumpkin seed; SES—sesame; RB—rice bran; ALM—almond; RPS—rapeseed; PNT—peanut; OL—olive; COC—coconut oil; EPO—evening primrose.

**Table 3:** Tocopherol and tocotrienol composition (mg/kg oil) in grape seed oils found in the literature [35,82,92,111].

	Study 1	Study 2	Study 3		
			France	Italy	Spain
Tocopherols					
α-T	47–56	86–244	18–229	14–160	tr–75
β-T	38–48	nd	nd–109	nd–133	nd–127
γ-T	17–29	3–28	nd–61	nd–119	nd–168
Δ-T	nd–3	tr–1	nd–47	nd	nd–69
Tocotrienols					
α-T3	216–278	69–319	nd–163	nd–352	nd–60
β-T3	-	4–18	nd–67	nd–22	nd–125
γ-T3	482–556	499–1575	nd–500	nd–785	nd–399
Δ-T3	13–17	6–18	nd	nd	nd–82

α-T: alpha-tocopherol; β-T: beta-tocopherol; γ-T: gamma tocopherol; Δ-T: delta tocopherol; α-T3: alpha-tocotrienol; β-T3: beta-tocotrienol; γ-T3: gamma-tocotrienol; Δ-T3: delta-tocotrienol; nd: not detected; tr: trace amount.

**Table 4:** Phytosterols (mg/kg oil) presents in grapes seed oil [109].

Phytosterols	Content (mg/kg Oil)
Cholesterol	nd–0.10
Cholestanol	nd
Brassicasterol	0.6–0.9
2,4 methylenecholesterol	nd–0.18
Campesterol	0.1–9.3
Campestenol	-
Stigmasterol	10.2–10.8
α-7 campesterol	0.16–0.27
α-52,3 stigmastadienol	-
Clerosterol	0.90–0.94
β-sitosterol	66.6–67.4
Sitostanol	3.92–4.70
α-5 avenasterol	1.98–2.09
α-52,4 stigmastadienol	0.41–0.47
α-7 estigmastenol	1.99–2.30
α-7 avenasterol	0.98–1.10

Abbreviations: nd, not determined.
